# Validation of a Simulated Commercial Plain Bagel Baking Process and Thermal Resistance Characterization of a 5-Strain Shiga Toxin-Producing *Escherichia coli* When Introduced via Flour

**DOI:** 10.3390/foods14071218

**Published:** 2025-03-31

**Authors:** Conor Hunt, Arshdeep Singh, Drushya Ramesh, Lakshmikantha H. Channaiah

**Affiliations:** Division of Food, Nutrition & Exercise Sciences, College of Agriculture, Food and Natural Resources, University of Missouri, Columbia, MO 65211-5200, USA

**Keywords:** bagels, Shiga toxin-producing *Escherichia coli*, validation, thermal resistance, baking, STEC, thermal inactivation

## Abstract

A study was conducted to validate the plain bagel baking process as an effective kill-step in controlling Shiga toxin-producing *Escherichia coli* (STEC) in the event of pre-baking contamination originating from flour. Unbleached bread flour was inoculated with five strains of STEC and dried back to its original water activity levels. The inoculated flour was used to prepare the bagel dough, proofed, boiled for 2 min, and baked at 232.2 °C (450 °F) for 14 min mimicking the commercial manufacturing process. Additionally, water activity (*a_w_*) and pH in plain bagels during baking, and thermal inactivation kinetics (*D*- and *z*-values) of STEC in plain bagel dough were studied. The results clearly demonstrated that baking plain bagels at 232.2 °C (450 °F) for 14 min will result in at least a >5 log reduction in the STEC population, thus providing an effective kill-step assuring the safety of the finished food products. The pH of plain bagels increased significantly from pre-proofed plain bagel dough to seven min into the baking process, reaching a final value of 5.83. The water activity of the crust and crumb portions of plain bagels was significantly different during the baking process. The *D*-values of STEC in plain bagels at, 56, 59, and 62 °C were 26.3 ± 1.55, 9.0 ± 0.27, and 2.50 ± 0.15 min with a *z*-value of 5.8 ± 0.16 °C.

## 1. Introduction

Flour is a minimally processed, low-moisture raw agricultural food ingredient that lacks a conducive environment for microbial growth. Although most pathogenic bacteria cannot grow in wheat flour, they can still survive and have the potential to cause foodborne illness outbreaks [1,2,3,4,5]. Pathogens such as *Salmonella*, and Shiga toxin-producing *Escherichia coli* (STEC) can remain viable in the flour for months to two years [1,2,3,4,5]. Furthermore, pathogens such as STEC can start to grow when water is added to flour to prepare various bakery food products [1,6,7]. STEC is considered one of the important foodborne pathogens, capable of causing severe illness through contaminated food products resulting in a life-threatening complication such as hemolytic uremic syndrome (HUS) [1,8,9]. The STEC has been associated with several foodborne illness outbreaks linked to flour and its derivatives in the United States [1]. In June 2016, the CDC (Centers for Disease Control and Prevention) announced a multistate outbreak of *E. coli* O121 linked to flour produced at a mill in Kansas City, MO. A total of 63 people were infected with the outbreak strains of STEC O121 or STEC O26 from 24 states with 17 people hospitalized, one affected patient developed hemolytic uremic syndrome, a type of kidney failure, and no deaths were reported [10]. The investigation indicated that flour produced at a mill in Kansas City, MO, was the likely source of this outbreak. In May 2019, the CDC reported another multistate outbreak of *E. coli* O26 investigation linked to flour. Several flour derivative products including bread flour, all-purpose flour, unbleached all-purpose flour, bread flour, and cookie and brownie mixes were recalled due to potential *E. coli* O26 contamination [11]. A total of 21 people were infected with the outbreak in nine states with three hospitalizations and no deaths were reported. The CDC’s epidemiologic and laboratory evidence indicated wheat flour was the likely source of this outbreak. Furthermore, in 2021, the CDC released an investigation report wherein *E. coli* O121 outbreak was linked to cake mix [1]. A total of 16 people were infected from 12 states with seven hospitalizations, one person developed a hemolytic uremic syndrome (HUS), and no deaths have been reported. The FDA’s (the U.S. Food and Drug Administration) traceback investigation revealed that sick people bought various brands of cake mixes. Due to multiple flour-related outbreaks, both the FDA and the CDC have advised consumers not to eat raw dough or batter without baking [12]. These outbreaks affect consumer confidence and cause significant economic losses to food manufacturers, besides affecting the lives of consumers [12,13,14]. Over the years, the milling industry has tried out new technologies such as the application of ozone [15], radio frequency heating [16], heat treatment of flour [17], etc., to decontaminate the flour during the milling process. However, the cost of implementing new technologies and their detrimental impact on flour baking qualities are some of the challenges that limit millers from manufacturing ready-to-eat flour products. In order to address the food safety risks in a farm-to-fork model, the FDA’s Food Safety Modernization Act (FSMA) mandates validation of preventive controls, such as baking or cooking, to control the potential foodborne pathogens including STEC to ensure the safety of finished food products [16,18]. The FDA’s Food Safety Modernization Act’s (FSMA) mandates validation requirement (21 CFR § 121.160) for all the registered food processors that manufacture human food products [19,20]. Specifically, the FDA’s Preventive Control for Human Food (PCHF) requirements requires that one must identify and implement effective preventive controls to provide assurances that any hazards requiring a preventive control will be significantly minimized or prevented and the food manufactured, processed, packed, or held by the facility will not be adulterated under section 402 of the Federal Food, Drug, and Cosmetic Act (FD&C Act) (21 U.S.C 342) or misbranded under section 403(w) of the FD&C Act (21 U.S.C. 343(w)) [20].

Bagels are a popular breakfast food in the United States and worldwide with a market value of USD 4.63 billion in 2023 [21]. Bagels are also a very popular breakfast or snack bakery product in Europe, Asia Pacific, Middle East, Africa, and Central and South America. Although the origins of bagels are unclear, it is believed that they were created in the 17th century in Poland as a response to anti-Semitic laws that prevented Polish Jews from baking bread [22]. A bagel is a doughnut-shaped yeast-leavened bread roll that is characterized by a crisp, shiny crust, a dense interior, and is shaped by hand into a torus or ring, boiled in water for a short period of time and then baked. This unique method of boiling and baking results in a dense, chewy, doughy interior with a browned and crisp exterior. The major ingredients used in bagel manufacturing are flour, yeast, salt, and sweetening. In 2020, an estimated 202 million Americans ate bagels at least once [22,23]. The survival of STEC in raw wheat flour for a prolonged period of time [2,3,24,25], its unusual heat-resistant characteristics in raw flour [3], and with over 60% of the U.S. population consuming bagels [21], call for a study to determine the effectiveness of the plain bagel baking process in controlling STEC in the event of pre-baking contamination originating from flour. This study focuses on plain bagels as they are the most popular bagels manufactured because they serve as a classic, versatile base that can be topped with almost anything, making them a favorite choice for breakfast and snacks. To the best of our knowledge, there have been no prior scientific studies on thermal inactivation of STEC in plain bagels. Therefore, a study was conducted to validate the effectiveness of the plain bagel baking process as an effective preventive control in controlling STEC assuring the safety of the finished food product. The main objective of this study is to validate the effectiveness of the plain bagel baking process (450 °F (232.22 °C) for 14 min) to control STEC contamination. Additionally, water activity, pH in plain bagels during baking, and thermal inactivation kinetics (*D*- and *z*-values) of STEC in plain bagel dough were studied.

## 2. Materials and Methods

### 2.1. Bagel Dough Preparation

The plain bagel recipe and the baking parameters were given by the American Institute of Baking International (AIB International, Manhattan, KS, USA). All the ingredients used in this study were purchased from a local grocery store in Columbia, MO, USA. Please refer to Table 1 for the bagel recipe. All ingredients were added to a sanitized metal bowl and then mixed at low speed for 7 min using a dough hook in a stand mixer (KitchenAid, Benton Harbor, MI, USA). Later, the dough was then weighed into two 30 g dough balls and twelve 85 g dough balls. The 85 g dough balls were then rolled out into ~21 cm in length and formed into the signature ring-shaped plain bagel with a hole in the center. One 30 g dough sample was rolled out and then taken as a pre-proof sample. The bagels were transferred to a cookie sheet with parchment paper, wrapped in plastic, and proofed for 25 min at 37 °C (98.6 °F). After proofing, the second 30 g sample was taken as the post-proof/0 sample. For the *D*- and *z*-value study, the inoculated dough was prepared as previously described but without yeast and was wrapped in a polyethylene-covered metal bowl and proofed at 37 °C (98.6 °F) for 25 min. Yeast was excluded from the formulation to prevent expansion of dough as well as formation of air pockets which would affect the temperature reading in the Thermal Death Time (TDT) disk thus preventing them from opening. Please refer to Table 2 for the recipe used to prepare plain dough for the *D*- and *z*-value study. Post proofing, 10 g of dough was then added to the TDT disk to determine the *D*- and *z*-values of STEC in bagel dough.

### 2.2. Experimental Design

Two separate studies were conducted to validate the plain bagel baking process to control STEC contamination and determine the heat resistance characteristics (*D*- and *z*-values) of STEC in plain bagel dough. The first study focused on validating a simulated bagel baking process where STEC was introduced via flour. Here the bagels were boiled for 2 min, and the bagels were flipped at 1 min to boil on both sides. After 2 min, the bagels were allowed to rest on a mesh wire rack for ~3 min to let excess water drain. After ~3 min, the plain bagels were baked at a temperature of 232 °C (450 °F) for 14 min followed by 15 min of ambient air cooling. There were a total of nine sampling points. viz., pre-proof dough, post-proof dough/0, 1 min of boiling, 2 min boiling, 3.5 min baking, 7 min baking, 10.5 min baking, and 14 min followed by a final sample taken after 15 min of ambient air cooling. Additionally, the water activity (*a_w_*) and pH were determined at these sampling points. This study employed a randomized complete block design with replications as blocks. Three independent replications were conducted, and the statistical significance was determined via one-way ANOVA using Minitab^®^.

The second study focused on determining the heat resistance characteristics (*D*- and *z*-values) of STEC in bagel dough. For this, we employed three temperatures viz., 56 °C, 59 °C, and 62 °C with the respective times of 0, 25, 50, 75, and 100 min; 0, 12, 24, 36, and 48 min; and 0, 2, 4, 6, and 8 min. Microsoft^®^ Excel 2024 was used to plot the linear regression graphs and statistical differences in STEC’s *D*- and *z*-values were determined using one-way ANOVA using Minitab^®^ 19 as the statistical software.

### 2.3. STEC Serovars

A total of five STEC strains were used in this study. Three strains were obtained from the American Type Culture Collection (ATCC^®^; Manassas, VA, USA). One from the United States Department of Agriculture (USDA) and another from the Centers for Disease Control and Prevention (CDC). Please refer to Table 3 for details. The STEC strains selected in this study were based on their association with foodborne illness outbreaks and/or recalls. The STEC strains were stored in Brain Heart Infusion (BHI) broth and stored at −20 °C (−4 °F). Before use, the STEC cultures were propagated in (BHI) broth for 24 h at 37 °C (98.6 °F) and then stored at 4 °C (39.2 °C).

### 2.4. Master Inoculum Preparation

To prepare the STEC master inoculum, BHI agar plates were inoculated with 250 µL of each STEC strain in duplicate (a total of 10 plates), and incubated at 37 °C (98.6 °F) for 24 h. Subsequently, the STEC plates were added with 1 mL of autoclaved 0.1% peptone water, and cells were harvested using a sterilized L-spreader. This process was repeated twice to harvest the maximum number of STEC cells. From respective STEC plates, the dislodged STEC cells along with 0.1% peptone water were then transferred to sterile 10 mL centrifuge tubes. Once all strains were harvested, the STEC inoculum from each tube was combined in a single 50 mL centrifuge tube to create a 5-strain STEC cocktail. The STEC cocktail was used to inoculate the flour used to prepare plain bagels.

### 2.5. Flour Inoculation

The flour inoculation was carried out as previously described (Channaiah et al., 2019) [26]. A total of 700 g of King Arthur^®^ unbleached bread flour was added to two sanitized plastic tubs (~38 × 26 × 7 cm, Rubbermaid Inc., Huntersville, NC, USA). A spray bottle nozzle calibrated to mist spray ~1 mL was attached to the 50 mL centrifuge tube containing the master inoculum. In a Class II, Type A2 Biological Safety Cabinet (LabConco Corporation, Kansas City, MO, USA), a total of 8 mL of STEC inoculum was mist inoculated into the flour contained in each plastic tub. The flour was then thoroughly mixed, and all lumps were broken up ensuring a uniform distribution of the STEC cells in the flour. Later, the flour was then dried in an incubator set at 37 ± 1 °C (98.6 ± 1.8 °F) for ~6 h until the *a_w_* level dried back to its pre-inoculation level. Once dried, the flour was then sealed in a plastic container and kept at room temperature (~21° [~70 °F]). The inoculated flour was used within 2 days of inoculation to prepare inoculated plain bagels. For the *D*- and *z*-value trials, 4 mL of STEC inoculum was mist inoculated into 200 g of flour, dried back to its pre-inoculation *a_w_* levels, and stored at room temperature (~21 °C (69.8 °F)) until use.

### 2.6. Plain Bagel Dough Boiling and Baking

A 6-channel temperature data logger (Super M.O.L.E.^®^ Gold 2, ECD, Milwaukie, OR, USA) was used to measure the internal temperature of the bagels every 1 s during boiling and baking. For this, five K-type thermocouples were inserted into the geometric center (as cold points) of the bagels randomly and the sixth thermocouple was used to measure the boiling water and/or oven air temperature. All the bagel doughs were placed into a metal cage (13.25″ × 8″) and lowered into a pot (measuring 14″ × 4″) containing boiling water. The bagels were boiled for one min (making sure they were not stuck to the bottom of the cage) and then flipped. The 1 min boil sample was taken at this time. The bagels were boiled for an additional min and the 2 min boil sample was taken at this time. At the completion of 2 min, all samples were removed and placed onto a metal rack to rest for 2.5 min ± 30 s. Later, the bagels were placed in the oven and baked at 232 °C (450 °F) for a total of 14 min. Samples were collected at 3.5, 7, 10.5, and 14 min for further analysis. After completing the 14 min baking process, all bagels were removed from the oven, and the final sample was collected following 15 min of ambient air cooling.

### 2.7. Water Activity (a_w_), pH, and Humidity Ratio

During the plain bagel baking process, the water activity (*a_w_*) was measured at each sampling point, including pre-proofed, post-proofed (0 min), after boiling for 1 and 2 min, and at baking intervals of 3.5, 7, 10.5, and 14 min. The final measurement was taken after 15 min of ambient air cooling (at 29 min), following the method described by Unger et al. (2021) [27]. For *a_w_* analysis, the plain bagel samples were placed into water activity cups, capped, and analyzed using a Novasina Labswift Portable water activity meter at (Novasina-AG, Lachen, Switzerland) 25 °C (77 °F) within ~30 min of removal of the samples. After 3.5 min into the baking, a noticeable crust formation was observed and two separate *a_w_* readings were collected for the crust and crumb potions of the bagels. A pH meter (FiveEasy F20 pH meter, Mettler-Toledo, Greifensee, Switzerland) was used to measure the pH of each plain bagel sample taken at various predetermined sampling points. The humidity inside the oven during baking was measured in a separate study using a Scorpion^®^ 2 Digital Humidity Sensor (Reading thermal, Sinking Spring, PA, USA). In this triplicate study, the humidity ratio (kg of moisture/kg of dry air) inside the oven during the plain bagel baking process was measured by placing the sensor inside the oven along with the plain bagel samples.

### 2.8. D- and z-Value Determination

For *D*- and *z*-value determination, the STEC master inoculum (~9 logs) was used to prepare the plain bagel dough. All five TDT disks (each containing 10 g plain bagel dough) and a T-type thermocouple (attached to one disk for internal temperature monitoring) were transferred to preset hot water baths at specified temperatures. The temperatures used in this study were 56 °C for 0, 25, 50, 75, and 100 min; 59 °C for 0, 12, 24, 36, and 48 min; and 62 °C for 0, 2, 4, 6, and 8 min. The TDT disks were placed into sterilized water baths (Precision CIR19, Thermo Fisher Scientific, Newington, NH, USA) heated to specified temperatures, and monitored in real time using a K-type thermometer (Fluke 51–2 Thermometer, Everett, WA, USA). TDT disks containing bagel samples were placed into the water bath in sets of 5 and the first sample (0 sample) was removed once the internal temperature of the disk reached the above target temperature. Once removed, the samples were immediately placed into an ice-water bath to prevent further thermal-killing.

### 2.9. Microbial Analysis

The enumeration of the STEC was carried out as previously described by Singh and Channaiah (2022) [28]. Samples collected at pre-proof, post-proof/0, 1 min boil, 2 min boil, 3.5 min bake, 7 min, 10.5 min, and 14 min as well as 15 min after ambient air cooling were analyzed for STEC survivability. Plain bagel samples (~30 g of each sample) were placed into Stomacher bags containing 50 mL of sterile 0.1% peptone water and stomached (Stomacher^®^ 400 Circulator, Worthing, West Sussex, UK) for 1 min at 300 rpm. After stomaching, the plain bagel samples were serially diluted in 9 mL of sterilized 0.1% peptone water and plated onto BHI agar. Plates were then inverted and incubated at 37° for 4 h where they were then overlaid (15 mL) with the selective media MacConkey agar and then continued incubating at 37° for another 20 h.

The analysis of the *D*- and *z*-values was performed by collecting the TDT disk from the ice bath and placing plain dough samples into stomacher bags with 10 mL of chilled sterilized 0.1% peptone water inside. Samples were then stomached (Stomacher^®^ 400 Circulator, Worthing, West Sussex, UK) for 1 min at 300 rpm, serially diluted onto the BHI agar, inverted and incubated at 37 °C (98.6 °F) for 4 h. Samples were then overlaid (~15 mL) with MacConkey agar and then further incubated at 37 °C (98.6 °F) for 20 h. After the incubation, the number of STEC cells was counted using a Reichert Quebec Darkfield Digital Colony Counter-110 V (Buffalo, New York, NY, USA) and reported as colony-forming units (CFU/g).

## 3. Results

### 3.1. Validation of Bagel Boiling/Baking to Control STEC

**Figure 1:**
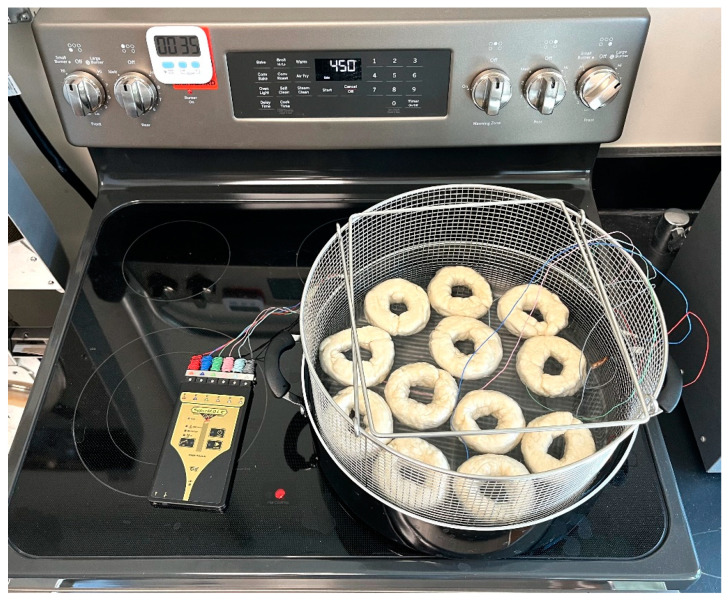
Plain bagel boiling setup.

**Figure 2:**
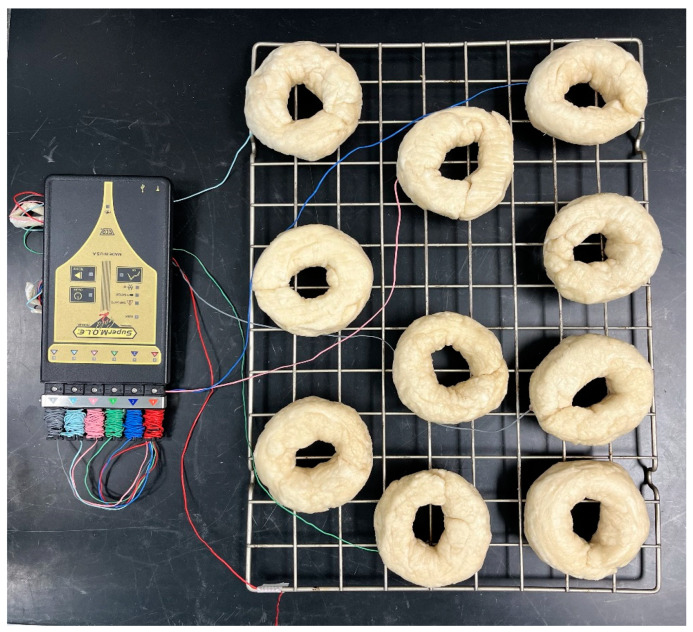
Plain bagel baking setup with K-type thermocouples.

**Figure 3:**
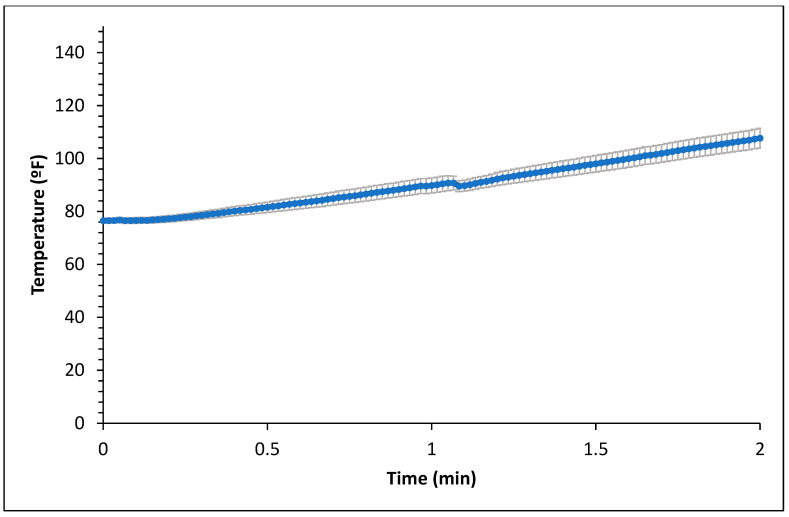
Mean internal temperature (±SE, *n* = 3) of plain bagels during boiling.

**Figure 4:**
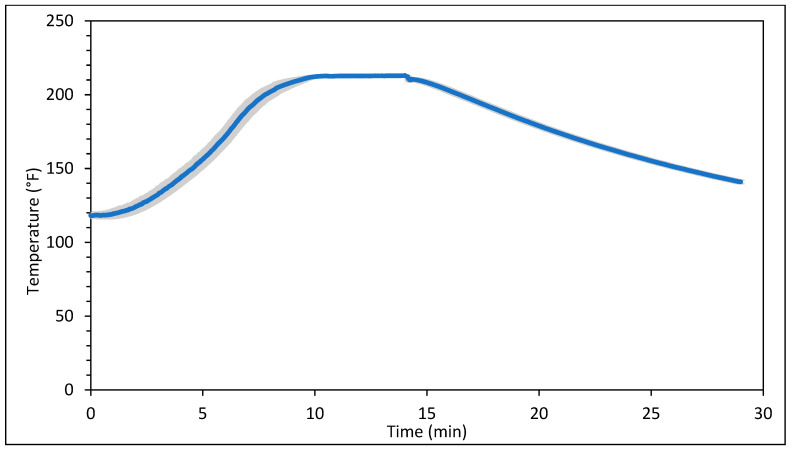
Mean internal temperature (±SE, *n* = 3) of plain bagels inoculated with STEC during 14 min of baking at 450 °F followed by 15 min of ambient air cooling.

**Figure 5:**
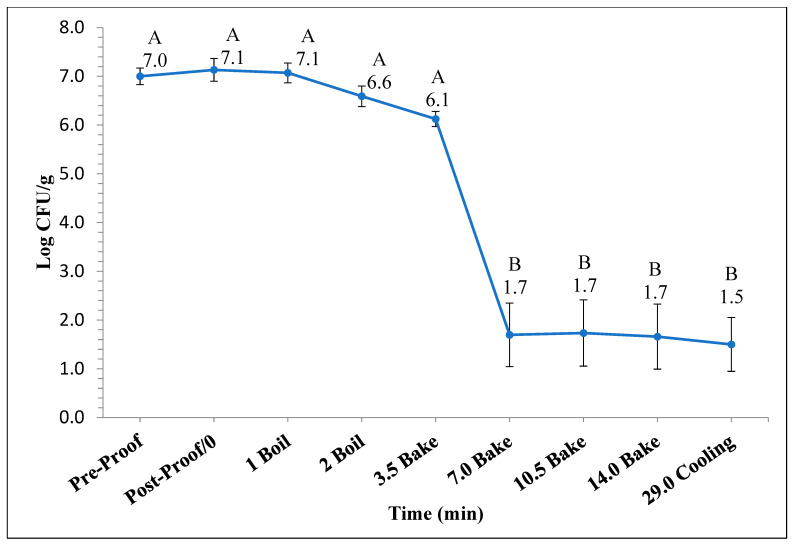
The mean log_10_ reduction (±SE, *n* = 3) in STEC population during 14 min of baking (450 °F) followed by 15 min of ambient air cooling when flour was used as the source of inoculum. Note: Data points with different letters are significantly different (*p* < 0.05). Detection limit is 0.4 log CFU/g.

**Figure 6:**
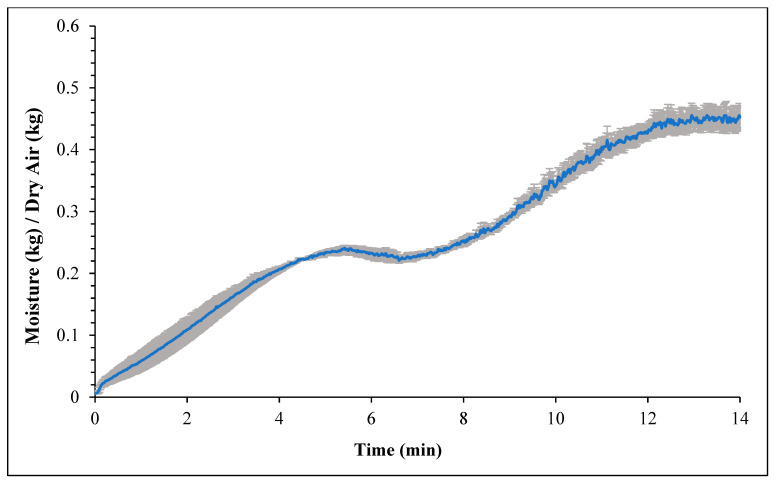
Mean humidity ratio (kg moisture/kg dry air) (±SE, *n* = 3) of the oven during 14 min plain bagel baking at 450 °F oven temperature.

**Figure 7:**
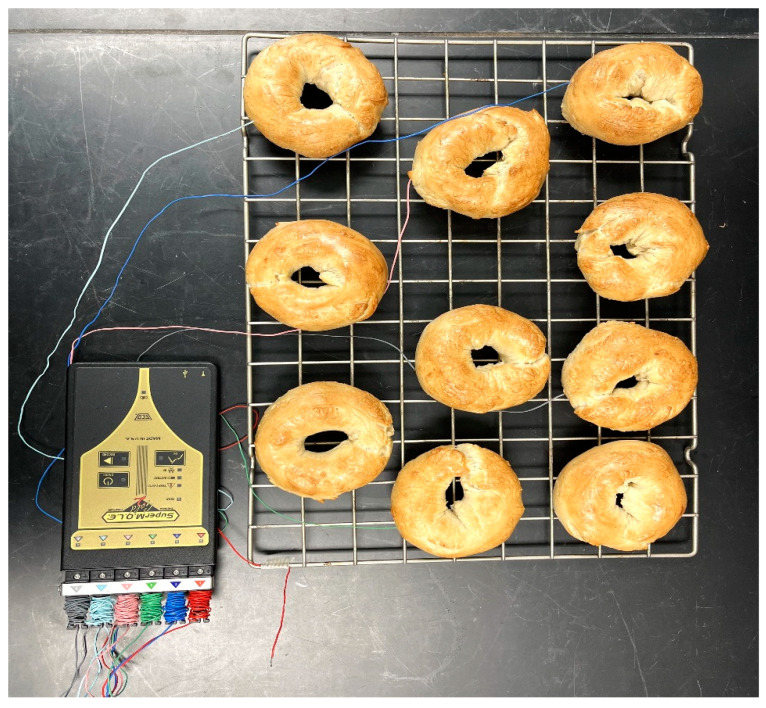
Plain bagels post baking with K-type thermocouples.

Figure 1 and Figure 2 represent the bagel boiling and baking setup. At the end of the boiling process, the mean internal temperature of bagels reached 107.7 ± 3.7 °C (225.8 ± 6.72 °F) as shown in Figure 3. The mean internal temperature of bagels at the start of the 14 min of baking process was 47.8 ± 0.8 °C (118.09 ± 1.51 °F) and reached a maximum of 212.8 ± 0.28 °F (100.45 ± 0.15 °C). However, at the end of 15 min of ambient air cooling, the mean internal temperature of plain bagels dropped to 60.5 ± 0.66 °C (140.9 ± 1.2 °F) (Figure 4). The STEC master inoculum used to inoculate bread flour to prepare plain bagels had an initial concentration of 11.3 ± 0.05 log CFU/g of STEC cells. After drying, the inoculated bread flour retained 5.90 ± 0.03 log CFU/g of STEC populations. The pre- and post-proof plain bagel dough retained 7.0 ± 0.16 and 7.1 ± 0.23 log CFU/g of STEC populations. The combination of 2 min of boiling paired with 14 min of baking and 15 min of ambient air cooling resulted in a reduction of 5.5 ± 0.074 CFU/g of STEC population (Figure 5)*.* A significant reduction of >4 log CFU/g in the STEC population was observed between 3.5 and 7 min into the baking. A detection limit of 0.4 log CFU/g was established in this study. As seen in Figure 6, the mean humidity ratio in the oven was 0.00549 ± 0.00018 kg moisture/kg air at the beginning of baking which increased to 0.45243 ± 0.01923 kg moisture/kg air at the end of baking. Figure 7 shows the mean humidity ratio of plain bagel samples post boiling and baking.

**Figure 8:**
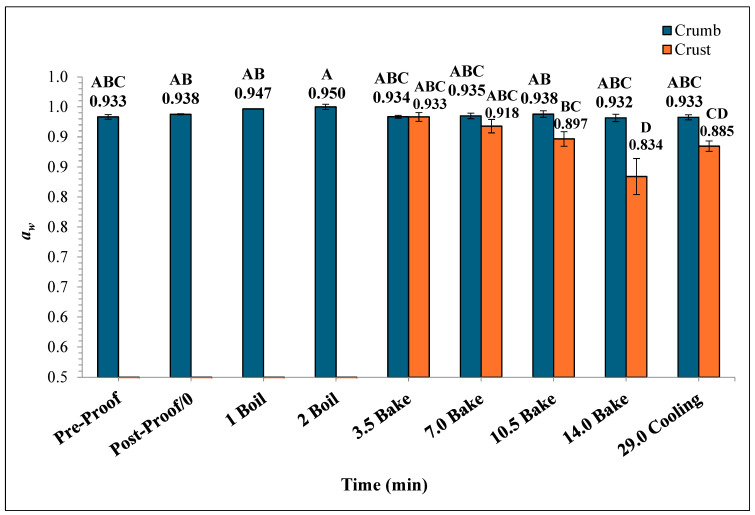
The water activity (*a_w_*) (±SE, *n* = 3) of the bagels was measured during 2 min of boiling, 14 min of baking, and at 29 min (after 15 min of ambient air cooling). Note: Data points with different letters are significantly different (*p* < 0.05).

In Figure 8, the mean crumb *a_w_* of the bagels showed no significant difference throughout the boiling and baking process, whereas the differences in mean crust *a_w_* were statistically significant. The crumb *a_w_* varied from 0.932 ± 0.006 to 0.950 ± 0.004 while the crust *a_w_* ranged from 0.834 ± 0.03 to 0.933 ± 0.007. As the baking process progressed, the mean crust *a_w_* of the plain bagels at the end of 7, 10.5, and 14 min of baking were 0.918 ± 0.011, 0.897 ± 0.012, 0.834 ± 0.030, respectively. During the ambient air-cooling period, the mean crumb *a_w_* of plain bagel samples reached 0.933 ± 0.009.

**Figure 9:**
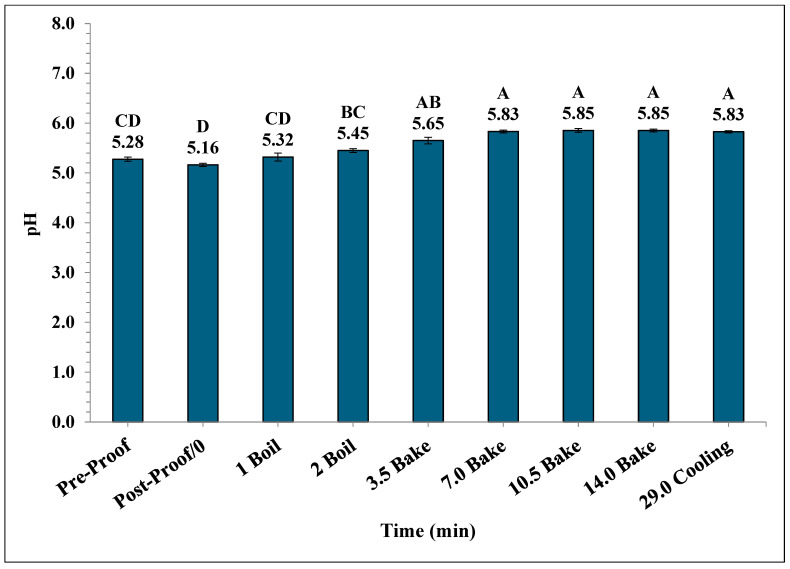
The pH (±SE, *n* = 3) of the bagels during 2 min of boiling, 14 min of baking, and after 15 min of ambient air cooling. Note: Data points with different letters are significantly different (*p* < 0.05).

A significant increase in the mean pH of the bagels occurred during the boiling and baking process. The mean pH in bagels varied from 5.28 ± 0.04 during pre-proofing to 5.83 ± 0.02 at the end of 15 min of ambient air cooling (Figure 9). Also, the mean pH of post-proof bagel samples was 5.16 ± 0.032 which is significantly lower compared to the mean pH of plain bagel samples collected at other pre-determined time intervals.

### 3.2. D- and z-Values of STEC in Bagel Dough

**Figure 10:**
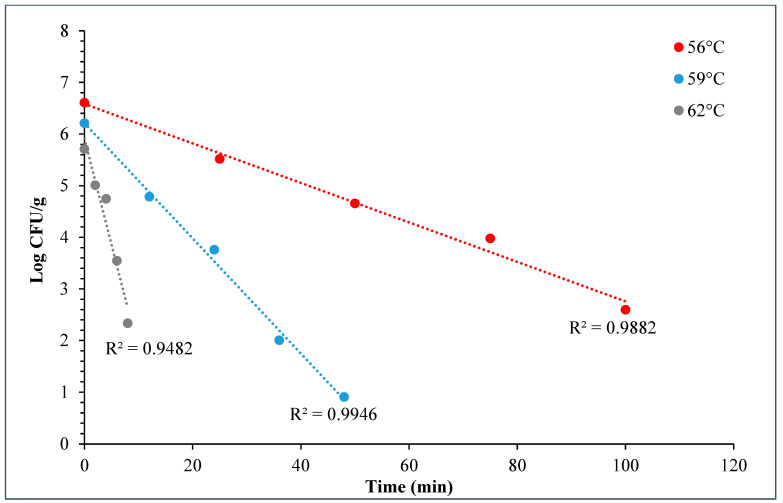
Graph showing thermal inactivation of 5-strain STEC populations (log CFU/g) vs. time (min) (±SE, *n* = 3) used for *D*-value calculation in bagels.

**Figure 11:**
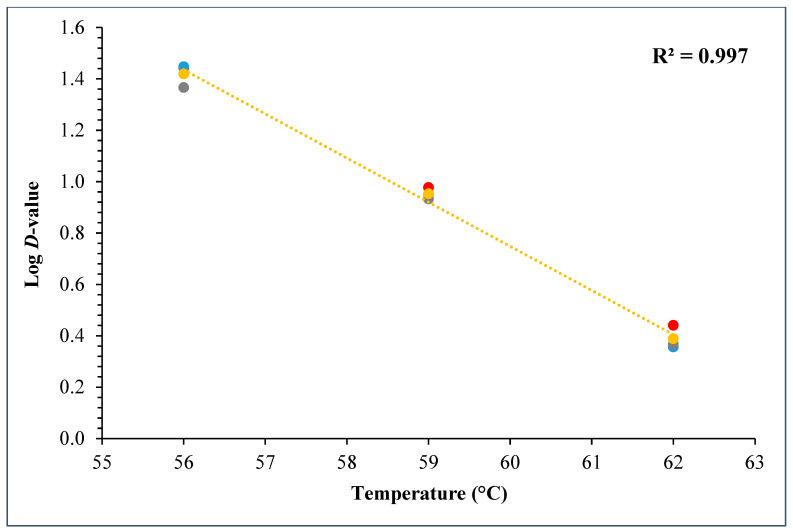
Graph showing log *D*-values (mean) versus temperature (°C) used for calculating *z*-values for the 5-strain STEC cocktail.

During the *D*- and *z*-value study, the master inoculum had an initial STEC concentration of 10.76 ± 0.11 log CFU/g. Whereas the inoculated flour retained 7.2 ± 0.10 log CFU/g STEC while the post-proof dough had 6.9 ± 0.17 log CFU/g of STEC cells. A linear regression plot was drawn by plotting the log population vs. time, wherein three different temperatures were used in calculating the *D*-value for this STEC cocktail. The *D*-values for 56 °C, 59 °C, and 62 °C were 28.3 ± 1.55, 9.0 ± 0.27, and 2.5 ± 0.15 min (Figure 10). The log of these *D*-values was taken and plotted against temperature (°C) to obtain the *z*-value of 5.8 ± 0.16 °C (Figure 11). Table 4 displays the calculated *D*- and *z*-values.

## 4. Discussion

Flour is a minimally processed raw agricultural product and thus requires thorough cooking before consumption of finished food products [10]. Despite the fact that flour is a low-water activity ingredient that generally does not favor bacterial growth, pathogenic microorganisms such as STEC can survive in flour for weeks in a desiccated state [10,29,30]. The STEC is known to cause ~265,000 infections in the United States annually, and it has been identified as one of the potential pathogens that can contaminate flour, leading to foodborne illness outbreaks [10].

The pH of plain bagels increased significantly from pre-proofed plain bagel dough samples to 7 min into the baking process, reaching a final value of 5.83. Similar trends have been reported by earlier studies [26,28,31]. Channaiah et al. (2017) reported that the pH of the muffin batter increased from 6.61 ± 0.12 to 7.49 ± 0.04 by the end of 21 min of baking [31]. In another study, Channaiah et al. (2019) reported that the pH of nut muffin batter increased from 6.50 ± 0.21 to 7.37 ± 0.05 at the end of 21 min of baking during the nut muffin baking process [26]. Singh et al. (2024) studied the validation of a simulated commercial English muffin baking process to control *Salmonella* and reported that the pH of English muffin samples increased from 5.01 ± 0.01 to 5.45 ± 0.05 by the end of the baking process [32].

The *a_w_* of the crust and crumb sections of plain bagels were significantly different during the baking process. This difference in *a_w_* in the crust and crumb of plain bagels could be attributed to the direct exposure of plain bagel crust to oven heating while baking. However, the *a_w_* of the crumb portion of plain bagels remained unchanged during the baking and cooling process (0.933 to 0.933). The drastic differences in the *a_w_* of the crust and crumb of plain bagels could be attributed to case hardening [32,33]. The case hardening is a phenomenon where the product’s outer layer dries much faster than the core or crumb portion during the baking process [33]. Similar trends were observed by earlier researchers. Singh et al. (2024) reported on the *a_w_* of the crust and crumb portions of English muffin samples during the English muffin baking process [32]. Singh et al. (2024) reported that the *a_w_* of crumb of the English muffin remained statistically unchanged with an initial value of 0.9470 to 0.9583 versus the crust samples which significantly decreased from 0.9431 to 0.9180 by the end of the 18 min baking process [32]. In general, the heat resistance and survival of *Salmonella* in low-moisture food products are greatly impacted by the water activity [34,35,36,37,38,39]. The significant influence of *a_w_* and other baking parameters on *Salmonella*’s heat resistance characteristics makes it hard to extrapolate the results of this study. For example, organic wheat flour demonstrated a *D*-value of 4.3 ± 0.02 at 0.73 water activity compared to a *D*-value of 7.3 ± 0.7 at 0.46 water activity at 80 °C (36).

Boiling plain bagels for 2 min resulted in a 0.4 log CFU/g reduction in the STEC population. At the end of the 1 and 2 min boiling of plain bagels, the mean internal temperature reached 89.81 ± 2.84 °F (32.11 ± 1.58 °C) and 107.69 ± 3.74 °F (42.05 ± 2.07 °C), respectively. The low reduction of the STEC population observed could be attributed to the short boiling step and low heat penetration in plain bagels.

The STEC population experienced a significant decrease after 7 min into the bagel baking process. A total reduction of 5.5 ± 0.074 log CFU/g in STEC populations were observed when plain bagels were boiled for 2 min followed by baking at 450 °F for 14 min. Various researchers demonstrated similar trends in STEC populations while validating baking as an effective kill-step involving various bakery products. Singh and Channaiah (2022) were able to demonstrate >5 log CFU/g reduction in STEC populations during the traditional crust pepperoni pizza baking process when baked at 500 °F (260 °C) for 12 min [28]. Michael et al. (2020) reported a >7 log CFU/g reduction in pathogenic *E. coli* O121 populations in muffins after baking at 375 °F (190.6 °C) for 17 min [7].

The *D*-values of STEC in plain bagels at, 56, 59, and 62 °C were 26.3 ± 1.55, 9.0 ± 0.27, and 2.50 ± 0.15 min with a *z*-value of 5.8 ± 0.16 °C. Other researchers also observed similar trends. Michael et al. (2020) studied the thermal inactivation parameters of a 4-strain cocktail of *E. coli* O121 in muffin batter [7]. Michael et al., 2020 reported that the *D*-values of *E. coli* O121 cocktail at 60, 65, and 70 °C were 42.0 ± 1.64, 7.5 ± 0.42, and 0.4 ± 0.03, respectively [7]. Whereas the *z*-value for the *E. coli* O121 cocktail in muffin batter was 5.0 ± 0.07 °C. Similarly, Singh and Channaiah (2022) studied the thermal inactivation parameters of a 7-strain cocktail of STEC in pizza dough and reported that the *D*-values at 55, 58, and 61 °C were 49.5 ± 4.10, 15.3 ± 0.68, and 2.8 ± 0.31 min [28]. The authors also reported the *z*-value as 4.8 ± 0.16 °C. The difference in the reported *D*-values in the above discussed studies when compared with the present study can be attributed to the use of different ingredients, processing parameters, and differences in intrinsic factors. The *z*-value reported in this study is similar to that reported by Michael et al. (2020) and Singh and Channaiah (2022), though the lower *z*-value could also be attributed to the varying protein and fat levels in the muffins, pizza, and plain bagels [7,28]. Additionally, the heat resistances of STEC strains used in each study may vary in different matrices, resulting in differences in the *D*- and *z*-values.

## 5. Conclusions

Effective implementation of validated preventive control is crucial to eliminating food safety hazards, assuring the safety of the finished food products and protecting consumers from foodborne illness outbreaks. This study validates and documents the first scientific evidence that boiling plain bagels for 2 min and baking them at 232.2 °C (450 °F) for 14 min serves as an effective kill-step by controlling the STEC population by a >5 log CFU/g. This study provides useful insight for food manufacturers to understand the effectiveness of thermal process steps in controlling foodborne pathogens such as STEC assuring the safety of the finished food products. The *D*- and *z*-values of STEC determined in plain bagel dough will help optimize the bagel baking process, thus achieving food safety. Furthermore, the *D*- and *z*-values of STEC determined in this study can be helpful in developing the STEC baking process predictive models for this matrix. The *D*- and *z*-values of STEC determined in this study are specific to the plain bagel recipe and baking parameters studied. It is also worth noting that the *D*- and *z*-values will vary if intrinsic properties such as fat, salt, sugar, *a_w_*, pH, etc., are changed. Therefore, to ensure food safety, it is highly recommended to conduct individual validation studies that are tailored to the recipe, baking parameters, and the target pathogen based on microbial risk analysis.

## Figures and Tables

**Table 1:** Recipe used for plain bagel validation study.

Ingredient	Weight (g)
Inoculated bread flour	700
Sugar	21
Salt	14
Dry yeast	5.6
Water	350

**Table 2:** Plain bagel recipe used for *D*- and *z*-value study.

Ingredient	Weight (g)
Inoculated bread flour	140
Sugar	4.2
Salt	2.8
Water	70

**Table 3:** List of STEC strains used in this study.

STEC	Strain	Origin
*E. coli* O157:H7	ATCC 43895	*E. coli* strain (CDC EDL 933) isolated from raw hamburger meat implicated in a hemorrhagic colitis outbreak
*E. coli* O157:H7	ATCC 43894	*E. coli* strain (CDC EDL 932) isolated from patient’s feces from outbreak of hemorrhagic colitis
*E. coli* O157:H7	C7927	*E. coli* strain isolated from a patient from an outbreak linked to contaminated apple cider (CDC)
*E. coli* O157:H7	MF 1847	Source: (USDA-FSIS). Originally isolated from hamburger meat, Food Microbiology Laboratory, Howard University, USA
*E. coli* O26:H11	ATCC BAA-2196	*E. coli* strain isolated from patient’s stool samples

**Table 4:** Mean (±SE, *n* = 3) *D*- (min) and *z*-values of STEC.

Items	Shiga Toxin-Producing *Escherichia coli*
56 °C	26.3 ± 1.55
59 °C	9.0 ± 0.27
62 °C	2.5 ± 0.15
*z*-value	5.8 ± 0.16

## Data Availability

The original contributions presented in the study are included in the article, further inquiries can be directed to the corresponding author.
